# Supplementary material for: Persistent intraocular Ebola virus RNA is associated with severe uveitis in a convalescent rhesus monkey
Source: Commun Biol. 2022 Nov 9;5:1204. doi: 10.1038/s42003-022-04158-2 (PMC9644391; doi:10.1038/s42003-022-04158-2)
Supplement: Supplementary file 2 — Supplementary Information [file 42003_2022_4158_MOESM2_ESM.pdf]

Supplementary Table 1. Euthanasia scores, EBOV nucleic acid (log_{10} EBOV glycoprotein [*GP*] gene equivalent [GEq]/mL) load in serum, and anti-EBOV *GP* IgG titers in serum measured over time. LOD; limit of detection.

Timepoint post-infection [a]	Euthanasia score (1 through 4)	EBOV *GP* GEq/mL (log_{10})	anti-EBOV *GP* IgG (titer log_{10})
Baseline	0	0	1.70 (<LOD)
0 AM	0		
0 PM	0	0	
1 AM	0		
1 PM	0		
2 AM	0		
2 PM	0		
3 AM	0		
3 PM	0		
4 AM	1		
4 PM	1	6.65	1.70 (<LOD)
5 AM	1		
5 PM	1		
5 LC	1		
6 AM	1		
6 PM	1		
6 LC	1		
7 AM	0		
7 PM	0	5.21	N/A
7 LC	0		
8 AM	0		
8 PM	1		
8 LC	1		
9 AM	2		
9 PM	2	7.23	1.70 (<LOD)
9 LC	1		
10 AM	1		
10 PM	1		
11 AM	1		
11 PM	1		
12 AM	1		
12 PM	1	4.48	3.43
13 AM	1		
13 PM	1		
14 AM	0	0	
14 PM	0	0	

21 AM	0	0	4.54
28 AM	0	0	4.53
37 AM	0	0	4.29
44 AM	0	0	4.25
51 AM	0	0	4.16
58 AM	0	0	4.07
65 AM	0	0	4.25
72 AM	0	0	4.16
79 AM	0	0	3.96
86 AM	0	0	3.85
99 AM	0	0	4.22

[a] Morning, AM; afternoon, PM; late check, LC

Supplementary Table 2. Clinical pathology parameters (platelets and aspartate aminotransferase [AST]) during acute Ebola virus (EBOV) infection and early convalescence. In-house reference intervals for Chinese rhesus are 201—467 x 10^3/µL and 22—46 U/L for platelets and AST, respectively.

Day post-infection	Platelets (x 10^3/µL)	AST (U/L)
0	474	30
4	329	39
7	423	34
9	292	116
12	202	63
21	492	25
28	447	28

Supplementary Table 3. External signs of uveitis recorded daily via cageside observation. Presence of signs is indicated with "X".

Day post-infection	Periorbital edema/asymmetry of eyes	Periorbital erythema	Anterior chamber fibrin/cloudiness/clot	Anisocoria
11				
12				
13				
14				
15				
16				
17				
18				
19				
20				
21	X			
22	X			
23	X	X		
24	X	X		
25	X	X		
26	X	X		
27	X	X		
28	X	X	X	
29	X	X	X	
30	X	X	X	
31			X	X
32				X
33				X
34				X
35				X
36				X
37				X
38				X
39				X
40				X
41				X
42				X
43				X
44				X

45				X
46				
47				X
48				X
49				
50				
51				
52				
53				
54				
55				
56				
57				
58				
59				
60				
61				
62				
63				
64				
65				
66				
67				
68				
69				
70				
71				
72				
73				
74				
75				
76				
77				
78				
79				
80				
81				
82				
83				

84				
85				
86				
87				
88				
89				
90				
91				
92				
93				
94				
95				
96				
97				
98				
99				

Supplementary Table 4. Eye globe volume measurements extrapolated from magnetic resonance (MR) images acquired on days 37, 51, 72 and 91 post-infection.

Day post-infection	Right eye (mL)	Left eye (mL)
37	4.13	3.46
51	4.14	3.72
72	4.08	3.73
91	4.09	3.83

Supplementary Figure 1. Cytology of the vitreous fluid collected from the right eye (OD) on day 99. Sample is highly cellular with multiple very large rafts of well-differentiated neuroretina on a bloody and proteinaceous background.

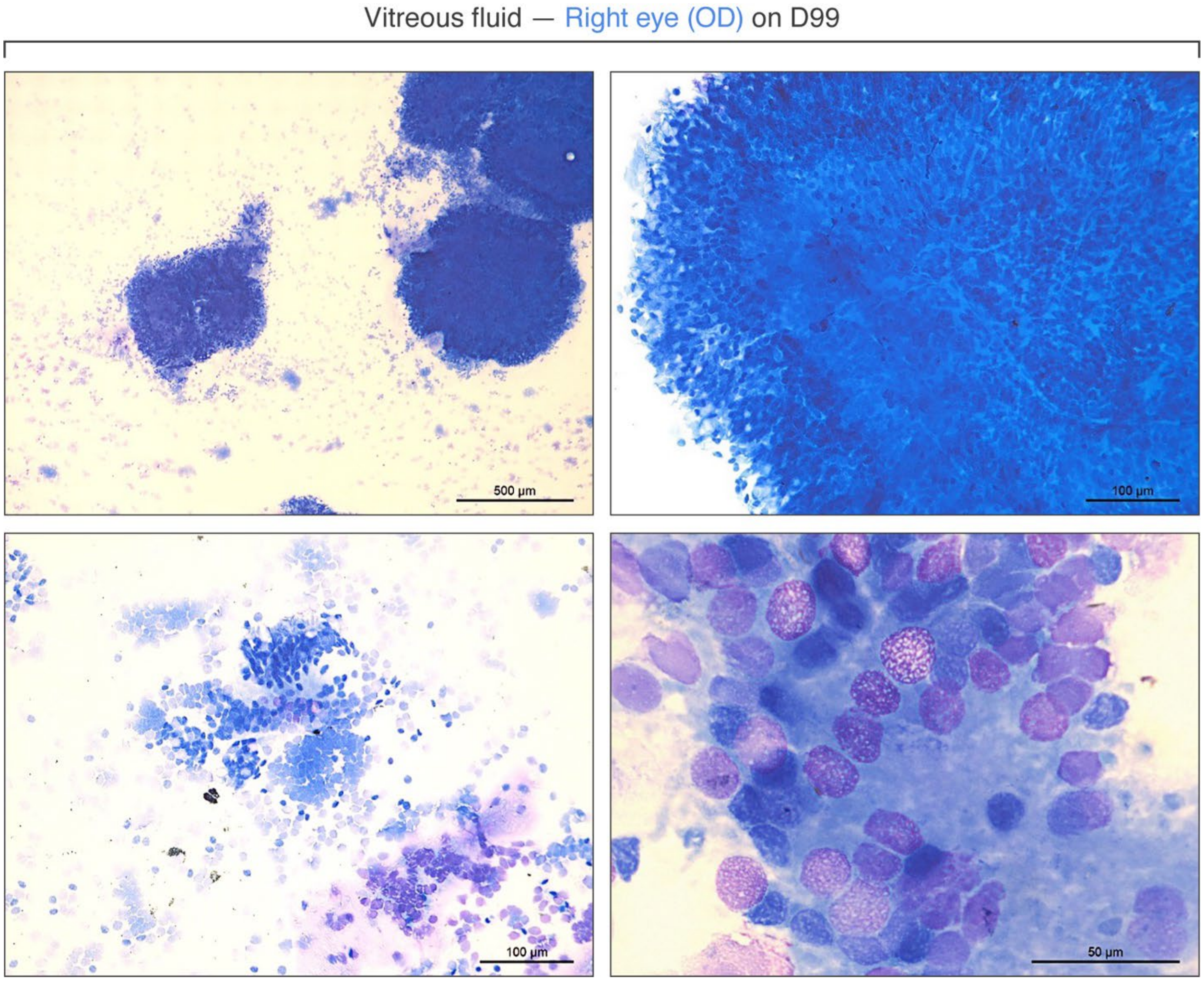

Supplementary Figure 2. Cytology of the vitreous fluid collected from the left eye (OS) on day 99.

a. Sample is extremely poorly cellular on a highly proteinaceous background. Cells present include isolated individual spindled cells with prominent cell processes (fibroblasts) as well as small round cells with condensed nuclear chromatin and a scant rim of cytoplasm (lymphocytes).

b. Negative *in situ* hybridization staining for genomic EBOV nucleic acid.

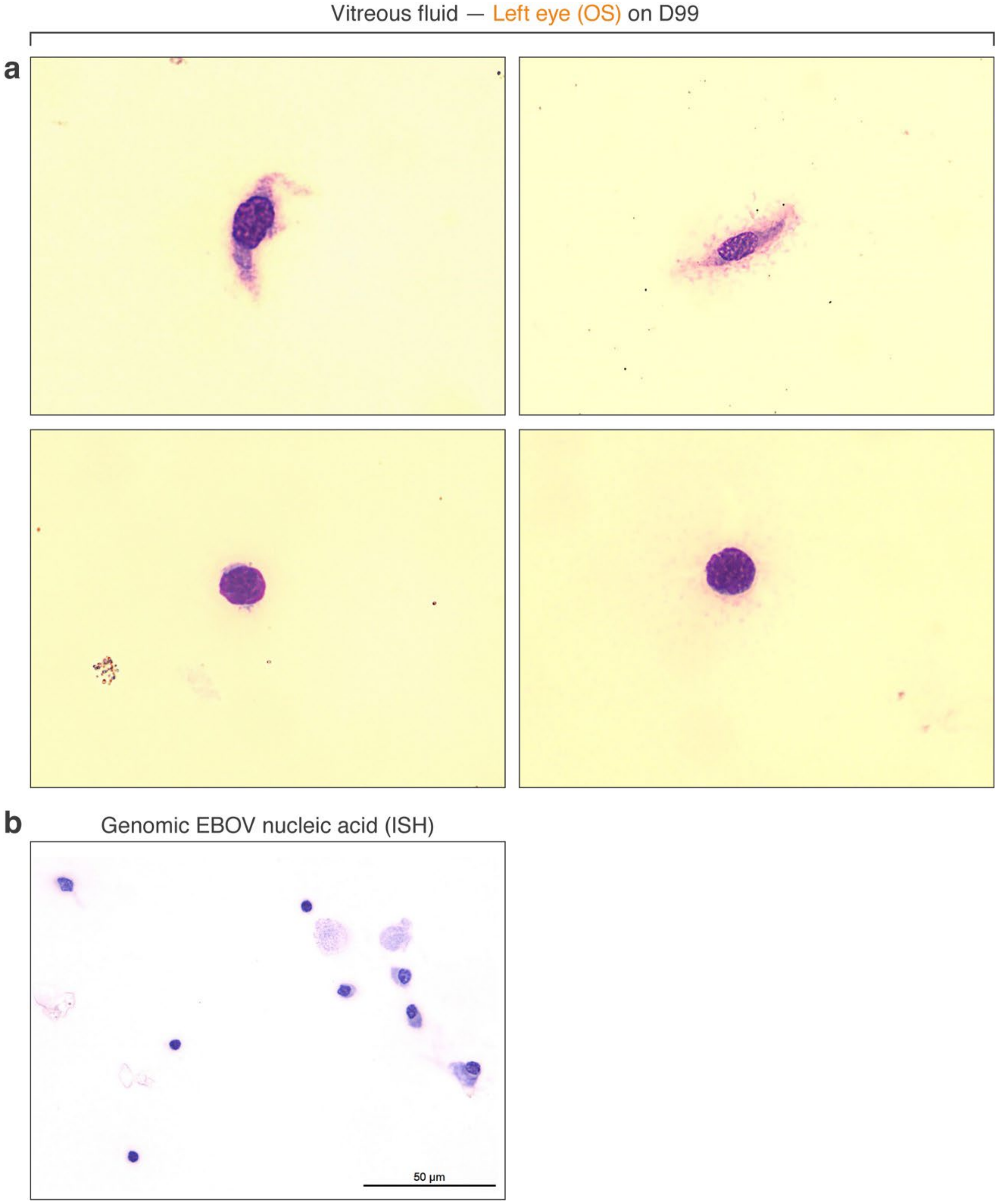

Supplementary Table 5. Total cell count in left (OS) and right (OD) eye vitreous humor.

Marker	OS, vitreous humor	OD, vitreous humor
CD45+ cells	137092.95	452.41
Total B cells	29.88	0.00
Naive B cells	0.00	0.00
Marginal-zone B cells	14.94	0.00
Memory B cells	14.94	0.00
Total CD3+ T cells	110110.62	159.67
Total CD4+ T cells	17196.38	0.00
Naive CD4+ T cells	44.82	0.00
Central memory CD4+ T cells	7709.24	0.00
Effector memory CD4+ T cells	9427.38	0.00
CD8+ T cells	79751.76	26.61
Naive CD8+ T cells	642.44	0.00
Central memory CD8+ T cells	27699.47	0.00
Effector memory CD8+ T cells	50961.64	26.61
Granulocytes	418.33	53.22
Monocytes	239.05	0.00
pDCs	0.00	0.00
mDCs	2241.06	0.00

Supplementary Table 6. Frequency of CD45+ cells (%) in left (OS) and right (OD) eye vitreous humor.

Marker	OS, vitreous humor	OD, vitreous humor
CD45+ Cells	1.00	
Total B Cells	0.02	0.00
Naive B Cells	0.00	0.00
Marginal-zone B Cells	0.01	0.00
Memory B Cells	0.01	0.00
Total CD3+ T Cells	80.32	35.29
Total CD4+ T Cells	12.54	0.00
Naive CD4+ T Cells	0.03	0.00
Central Memory CD4+ T Cells	5.62	0.00
Effector Memory CD4+ T Cells	6.88	0.00
CD8+ T Cells	58.17	5.88
Naive CD8+ T Cells	0.47	0.00
Central Memory CD8+ T Cells	20.20	0.00
Effector Memory CD8+ T Cells	37.17	5.88
Granulocytes	0.31	11.76
Monocytes	0.17	0.00
pDCs	0.00	0.00
mDCs	1.63	0.00

Supplementary Figure 3. Gating used for flow cytometry analysis.

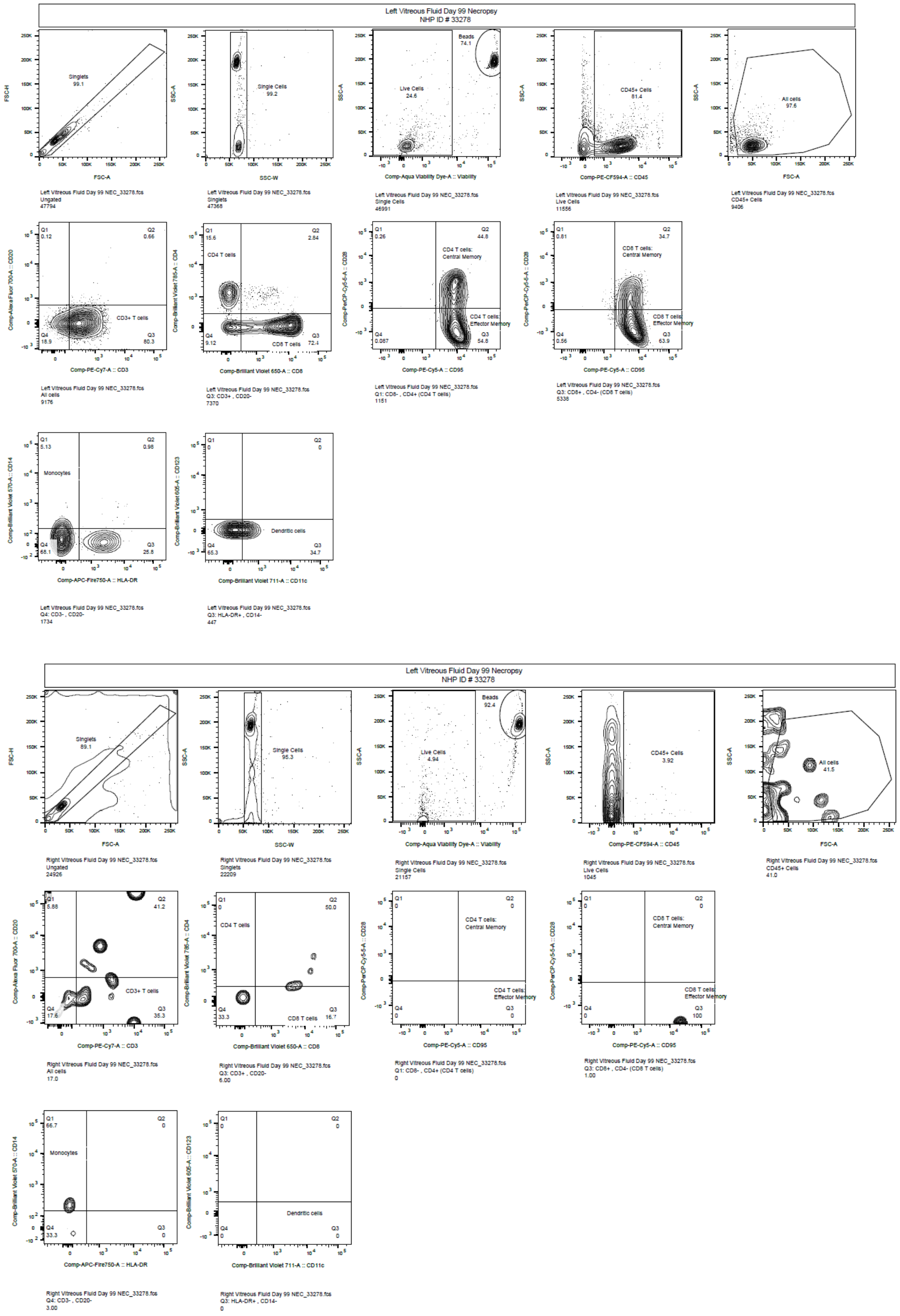

Supplementary Table 7. List of antibodies and reagents used for flow cytometry analysis of cells in left (OS) and right (OD) eye vitreous humor.

Antibody/ Reagent	Company	Catalog #	Lot #
Live/DEAD Fixable Aqua Dead Cell Stain Kit	Molecular Probes	L34957	2008162
CD27 BV421	Biolegend	356418	8265880
CD14 BV570	Biolegend	301832	8245763
CD123 BV605	BD Biosciences	564197	8222563
lgD FITC	Southern Biotech	2030-02	02016-S277B
CD197(CCR7) PE	BD Biosciences	560765	9025622
CD8 BV650	Biolegend	344730	8274094
CD4 BV786	BD Biosciences	563914	8316948
CD20 AF700	BD Biosciences	560631	8215552
CD28 PerCP-Cy5.5	Biolegend	302922	8275635
CD45 PE-CF594	BD Biosciences	562394	8240808
CD95 PE-Cy5	Biolegend	305610	B266014
CD3 PE-Cy7	BD Biosciences	557749	8208643
CD11c BV711	Biolegend	301630	B278037
HLA-DR APC	Biolegend	307658	B280512

Supplementary Table 8. Proinflammatory cytokines in left (OS) and right (OD) eye vitreous humor from the rhesus monkey survivor on day 99 compared to vitreous humor from an EBOV-naïve rhesus monkey (control).

Sample	control	OD	OS
GM-CSF	0.80	0.87	1.28
TGF-alpha	0.20	0.30	0.23
G-CSF	9.77	27.54	58.37
IFN-gamma	0.00	0.00	3.92
IL-2	10.61	15.16	29.30
IL-10	0.00	12.88	37.62
IL-15	5.33	4.96	9.49
sCD40L	0.00	0.00	34.41
Il-17a	0.00	0.00	0.00
IL-1ra	54.06	25.45	18.52
IL-13	0.00	0.00	0.00
IL-1b	0.00	0.00	0.15
IL-4	0.00	0.00	0.00
IL-5	0.00	0.00	0.00
IL-6	0.00	0.70	16.86
IL-8	0.00	1.26	21.19
MIP-1a	2.65	0.00	11.56
MCP-1	344.93	468.81	1129.73
TNF-alpha	0.00	0.00	3.69
MIP-1b	0.00	0.00	0.00
IL-12/23(p40)	0.00	0.00	8.92
VEGF	0.00	3.74	142.17
IL-18	0.00	0.00	0.00

Supplementary Figure 4. Normal histopathology of the right eye (OD).

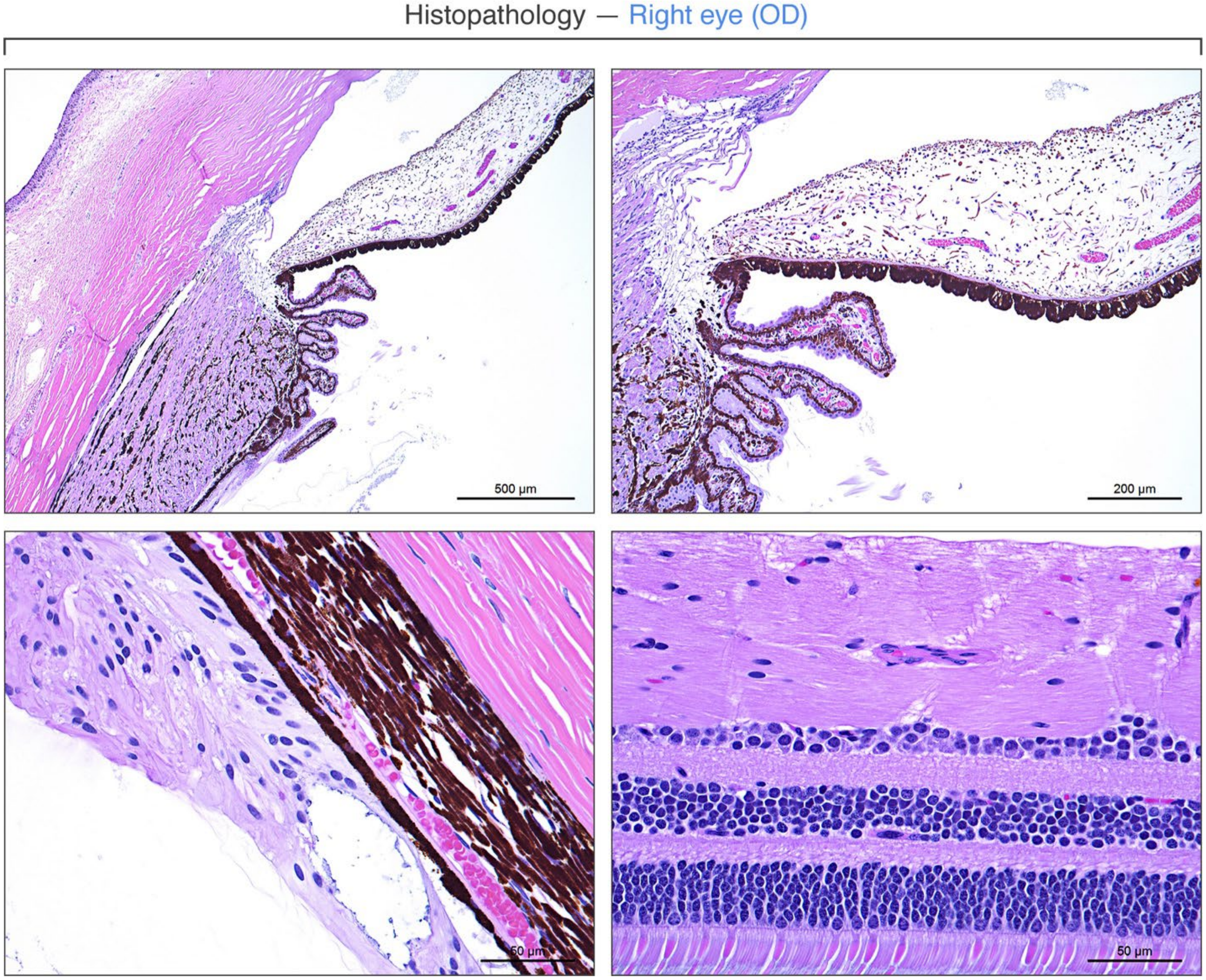

Supplementary Figure 5. Two very small foci of cataractous change (area within dotted line) in the posterior lateral subcapsular lens of the left eye (OS).

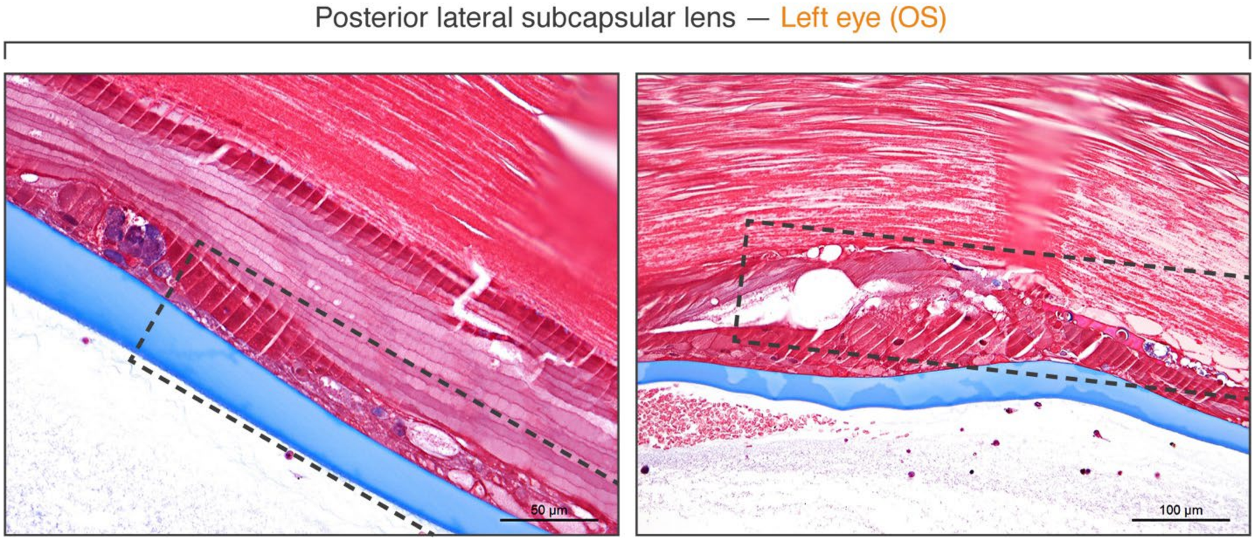

Supplementary Figure 6. Negative anti-EBOV protein (VP) 40 immunohistochemistry staining of left eye (OS).

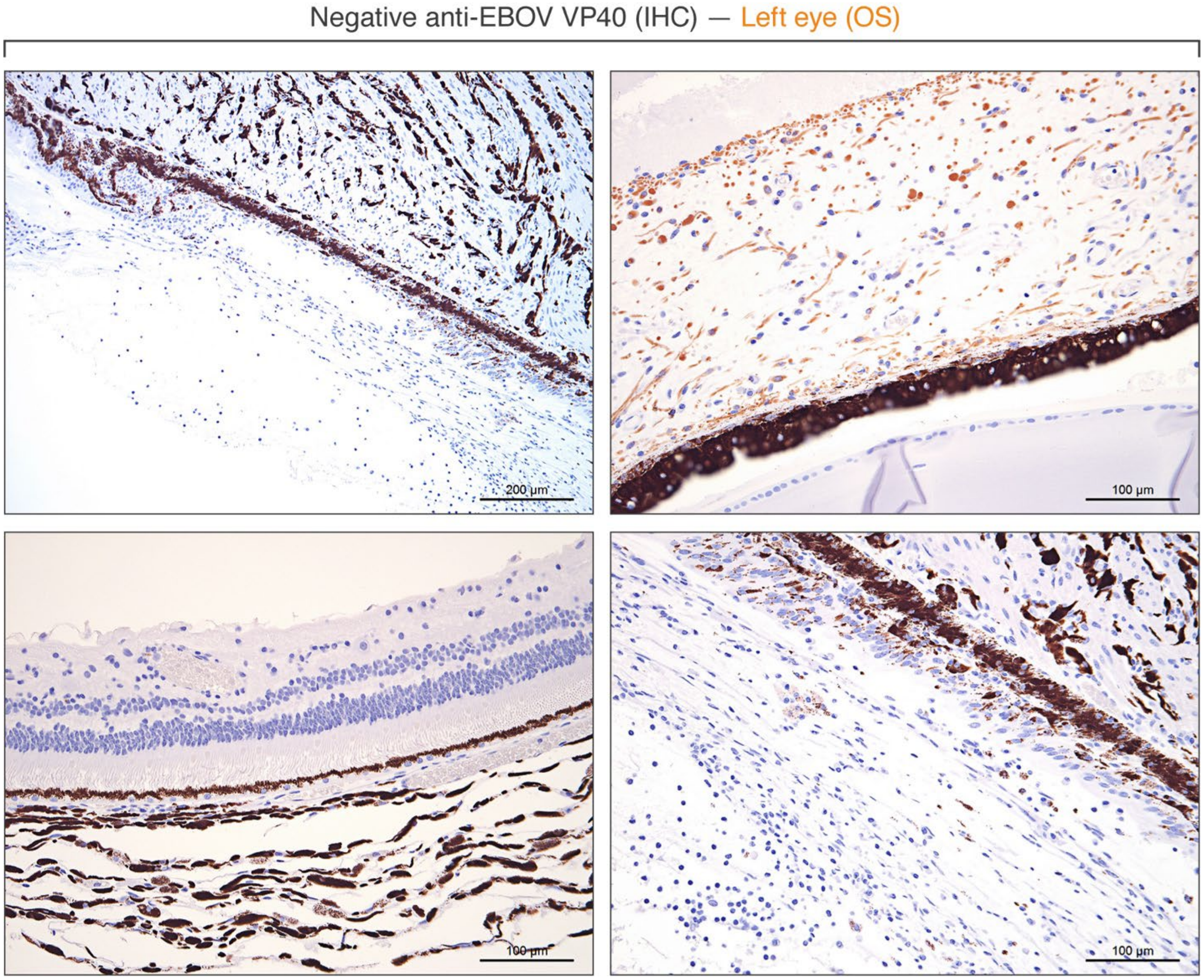

Supplementary Figure 7. Negative anti-EBOV glycoprotein (GP) immunohistochemistry staining of left eye (OS).

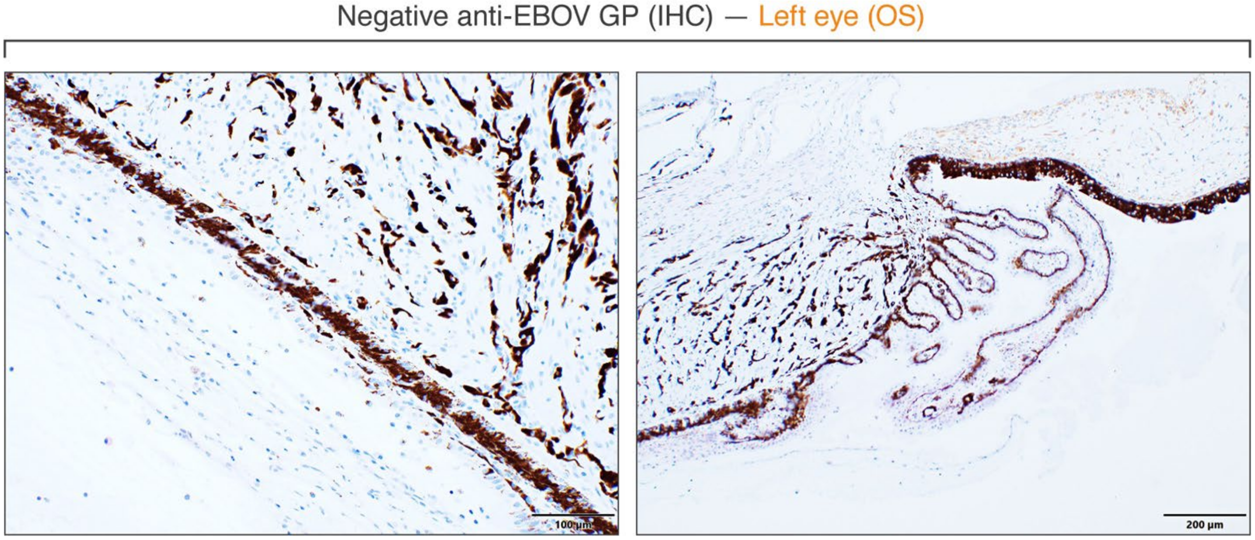

Supplementary Figure 8. Negative genomic EBOV nucleic acid *in situ* hybridization staining of left eye (OS).

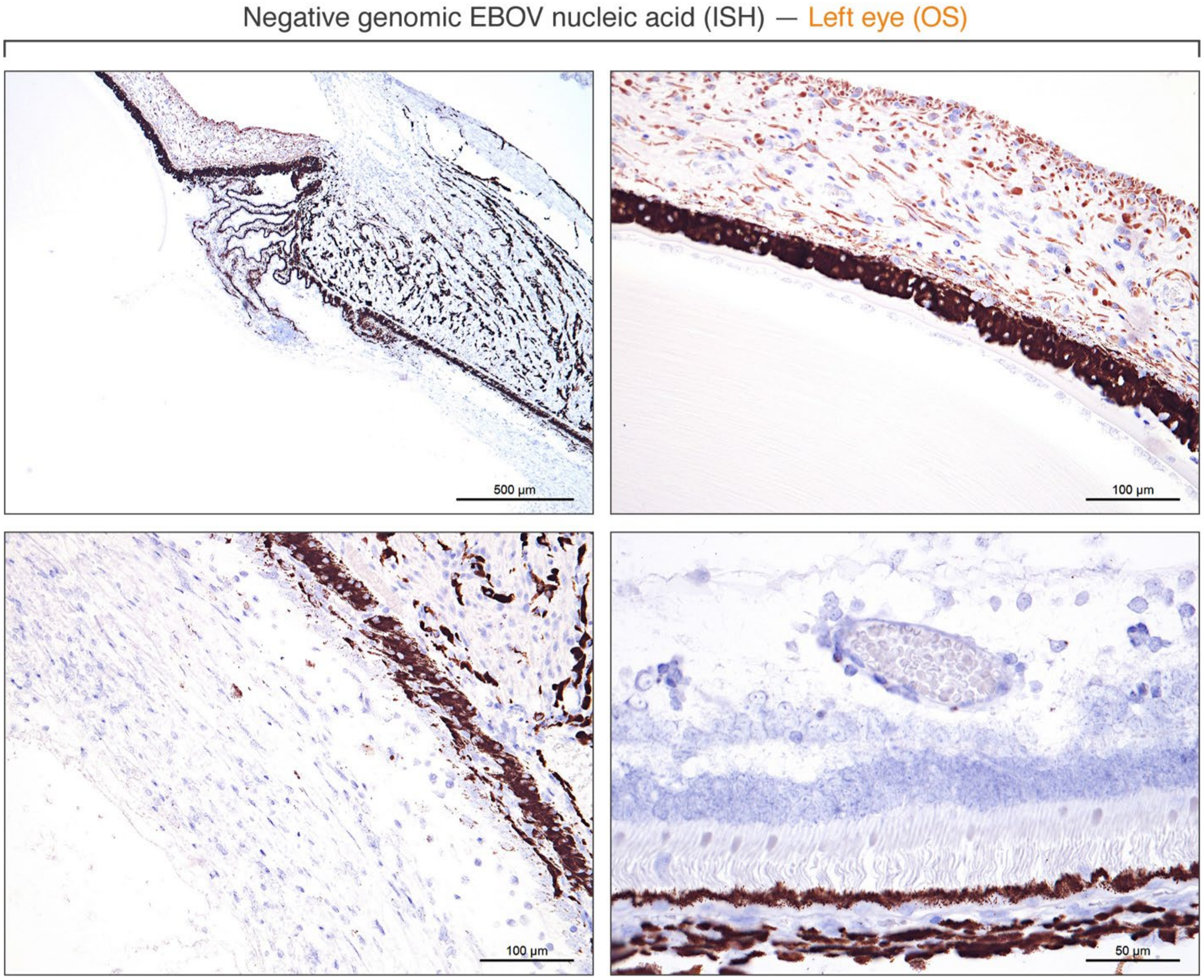

Supplementary Table 9. List of material references.

**Animal husbandry and products used for procedures**	
High Protein Monkey Diet	No. 5045, LabDiet, St. Louis, MO, USA
Ketamine HCl	at 15 mg/kg; KetaThesia, Henry Schein, Melville, NY, USA
9.20.1C3 anti-EBOV $GP_{1,2}$ antibody	Zalgen Labs, Germantown, MD, USA
1 % tropicamide solution	Tropicamide Mydriacyl eye drop, Alcon, Fort Worth, TX, USA
Tono-Pen Avia Vet applanation tonometer	Dan Scott & Associates, Westerville, OH, USA
Pictor Plus retinal camera	Volk Optical, Mentor, OH, USA
Pentobarbital sodium	Fatal Plus Solution; Vortech Pharmaceuticals, Dearborn, MI, USA

**Virology**	
Ebola virus/H. sapiens-tc/COD/1995/Kikwit-9510621 (EBOV)	NR-50306, BEI Resources, Manassas, VA, USA
Grivet (*Chlorocebus aethiops*) Vero E6 cells	BEI Resources, Manassas, VA, USA
Bead Ruptor Elite bead mill homogenizer	5.00 m/s for 90 s; Omni International, Kennesaw GA, USA
TRIzol LS	Thermo Fisher Scientific, Waltham, MA, USA
QIAamp Viral RNA Mini Kit	QIAGEN, Germantown, MD, USA
BEI Resources Critical Reagents Program EZ1 RT-PCR (TaqMan) assay kit	Trombley, A. R. *et al.* Comprehensive panel of real-time TaqMan^{TM} polymerase chain reaction assays for detection and absolute quantification of filoviruses, arenaviruses, and New World hantaviruses. *Am J Trop Med Hyg* **82**, 954-960, doi:10.4269/ajtmh.2010.09-0636 (2010).
Major groove binder (MGB) RT-PCR (TaqMan) assay	Trombley, A. R. *et al.* Comprehensive panel of real-time TaqMan^{TM} polymerase chain reaction assays for detection and absolute quantification of filoviruses, arenaviruses, and New World hantaviruses. *Am J Trop Med Hyg* **82**, 954-960, doi:10.4269/ajtmh.2010.09-0636 (2010).
ABI 7500 FastDx Real-Time PCR instrument	Applied Biosystems, Thermo Fisher Scientific, Waltham, MA, USA

**Immunology**	
LSRII Fortessa instrument	BD Biosciences, Franklin Lakes, NJ, USA

Luminex FLEXMAP 3D instrument	Luminex Corporation, Austin, TX, USA
MILLIPLEX MAP Non-Human Primate Cytokine Magnetic Bead Panel	Millipore Sigma, PCYTMG-40K-PX23, Burlington, MA, USA
Rhesus Monkey Immunoglobulin G ELISA kit	Molecular Innovations, Novi, MI, USA
anti-Ebola virus glycoprotein ($GP_{1,2}$) immunoglobulin G ELISA assay	Rudge, T. L., Jr. et al. Development, qualification, and validation of the Filovirus Animal Nonclinical Group anti-Ebola virus glycoprotein immunoglobulin G enzyme-linked immunosorbent assay for human serum samples. PLoS ONE 14, e0215457, doi:10.1371/journal.pone.0215457 (2019).

**Pathology**	
Mouse anti-EBOV matrix protein (VP40) antibody	at 1:1,500; 3G5, cat# 0201-016; IBT Bioservices, Rockville, MD, USA
Rabbit anti-EBOV glycoprotein ($GP_{1,2}$) antibody	at 1:14,000; cat# 0301-015; IBT Bioservices, Rockville, MD, USA
Mouse anti-CD4[BC/1F6] antibody	at 1:80; cat# CM153B; Biocare Medical, Pacheco, CA, USA
Rabbit anti-CD8 antibody	at 1:1500; cat# CM154; Biocare Medical, Pacheco, CA, USA
Rabbit anti-CD38 antibody	at 1:650; cat# LS-A9696; LSBio, Seattle, WA, USA
Mouse anti-CD68 antibody	at 1:500; cat# NBP-74570; Novus Biologicals, Centennial, CO, USA
RNAscope® 2.5 HD RED reagent kit	cat# 322360; Advanced Cell Diagnostics, Newark, CA, USA
EBOV-*VP40* (Genomic) RNA probe	cat# 507141; Advanced Cell Diagnostics, Newark, CA, USA
EBOV-*VP35* (Antigenomic or replicative intermediate) RNA probe	cat# 527491; Advanced Cell Diagnostics, Newark, CA, USA
Masson's Trichrome with Aniline Blue Stain	cat# 9179A; Newcomer Supply, Middleton, WI, USA
Warp red chromogen	cat# SK-5105; Vector Laboratories, Burlingame, CA, USA
Hematoxylin counterstain	cat# 7211; Richard-Allan Scientific-Thermo Fisher Scientific, Waltham, MA, USA

**Software**	
BD FACS Diva software version 6.1.3	BD Biosciences, Franklin Lakes, NJ, USA
FlowJo software version 10	FlowJo, Ashland, OR 97520, USA
Medical Image Merge (MIM) software version 6.9	Cleveland, OH, USA
GraphPad software version 8.4.2	Prism, La Jolla, CA, USA

Adobe Illustrator 25.4.8	Adobe, San Jose, CA 95110, USA

**Regulatory approvals**	
Public Health Service (PHS) Assurance for Laboratory Animal Welfare	D16-00602
United States Department of Agriculture (USDA)	51-F-0016
Association for Assessment and Accreditation of Laboratory Animal Care	777
NIAID DCR Animal Care and Use Committee (ACUC) animal study protocol	IRF 033E
