# Supplementary material for: Persistent intraocular Ebola virus RNA is associated with severe uveitis in a convalescent rhesus monkey
Source: Commun Biol. 2022 Nov 9;5:1204. doi: 10.1038/s42003-022-04158-2 (PMC9644391; doi:10.1038/s42003-022-04158-2)
Supplement: Supplementary file 3 — Reporting Summary [file 42003_2022_4158_MOESM3_ESM.pdf]

nature portfolio

Corresponding author(s): Gabriella Worwa, Ian Crozier

Last updated by author(s): 10/04/2022

Reporting Summary

Nature Portfolio wishes to improve the reproducibility of the work that we publish. This form provides structure for consistency and transparency in reporting. For further information on Nature Portfolio policies, see our Editorial Policies and the Editorial Policy Checklist.

Statistics

For all statistical analyses, confirm that the following items are present in the figure legend, table legend, main text, or Methods section.

n/a	Confirmed	
☒	☐	The exact sample size (*n*) for each experimental group/condition, given as a discrete number and unit of measurement
☒	☐	A statement on whether measurements were taken from distinct samples or whether the same sample was measured repeatedly
☒	☐	The statistical test(s) used AND whether they are one- or two-sided<br>*Only common tests should be described solely by name; describe more complex techniques in the Methods section.*
☒	☐	A description of all covariates tested
☒	☐	A description of any assumptions or corrections, such as tests of normality and adjustment for multiple comparisons
☒	☐	A full description of the statistical parameters including central tendency (e.g. means) or other basic estimates (e.g. regression coefficient) AND variation (e.g. standard deviation) or associated estimates of uncertainty (e.g. confidence intervals)
☒	☐	For null hypothesis testing, the test statistic (e.g. *F*, *t*, *r*) with confidence intervals, effect sizes, degrees of freedom and *P* value noted<br>*Give P values as exact values whenever suitable.*
☒	☐	For Bayesian analysis, information on the choice of priors and Markov chain Monte Carlo settings
☒	☐	For hierarchical and complex designs, identification of the appropriate level for tests and full reporting of outcomes
☒	☐	Estimates of effect sizes (e.g. Cohen's *d*, Pearson's *r*), indicating how they were calculated

Our web collection on statistics for biologists contains articles on many of the points above.

Software and code

Policy information about availability of computer code

Data collection	Medical Image Merge (MIM) software version 6.9 (Cleveland, OH, USA)
Data analysis	BD FACS Diva software version 6.1.3 (BD Biosciences, Franklin Lakes, NJ, USA) was used for collection of raw flow cytometry data. FlowJo software version 10 (FlowJo, Ashland, OR 97520, USA) was used for analysis of flow cytometry data. Medical Image Merge (MIM) software version 6.9 (Cleveland, OH, USA) was used for analysis of MR images. Initial graphs were generated using GraphPad software version 8.4.2 (Prism, La Jolla, CA, USA) and final artwork was created in Adobe Illustrator 25.4.8. (Adobe, San Jose, CA 95110, USA).

For manuscripts utilizing custom algorithms or software that are central to the research but not yet described in published literature, software must be made available to editors and reviewers. We strongly encourage code deposition in a community repository (e.g. GitHub). See the Nature Portfolio guidelines for submitting code & software for further information.

Data

Policy information about availability of data

All manuscripts must include a data availability statement. This statement should provide the following information, where applicable:

- Accession codes, unique identifiers, or web links for publicly available datasets
- A description of any restrictions on data availability
- For clinical datasets or third party data, please ensure that the statement adheres to our policy

Data generated or analyzed during this study are included in this article and its supplementary files. All other source data are available from the corresponding authors on reasonable request.

Human research participants

Policy information about studies involving human research participants and Sex and Gender in Research.

Reporting on sex and gender	N/A
Population characteristics	N/A
Recruitment	N/A
Ethics oversight	N/A

Note that full information on the approval of the study protocol must also be provided in the manuscript.

Field-specific reporting

Please select the one below that is the best fit for your research. If you are not sure, read the appropriate sections before making your selection.

☒ Life sciences ☐ Behavioural & social sciences ☐ Ecological, evolutionary & environmental sciences

For a reference copy of the document with all sections, see nature.com/documents/nr-reporting-summary-flat.pdf

Life sciences study design

All studies must disclose on these points even when the disclosure is negative.

Sample size	The report details unusual findings in one animal (n=1).
Data exclusions	N/A
Replication	N/A
Randomization	Assignment of animal to treatment groups prior to developing uveitis occurred in a randomized way.
Blinding	All staff was blinded to the type of therapeutic received. Additional blinding was not possible as the report describes a clinical manifestation that was visible to staff handling the animal.

Reporting for specific materials, systems and methods

We require information from authors about some types of materials, experimental systems and methods used in many studies. Here, indicate whether each material, system or method listed is relevant to your study. If you are not sure if a list item applies to your research, read the appropriate section before selecting a response.

Materials & experimental systems

n/a	Involved in the study	
☐	☒	Antibodies
☐	☒	Eukaryotic cell lines
☒	☐	Palaeontology and archaeology
☐	☒	Animals and other organisms
☒	☐	Clinical data
☒	☐	Dual use research of concern

Methods

n/a	Involved in the study	
☒	☐	ChIP-seq
☐	☒	Flow cytometry
☐	☒	MRI-based neuroimaging

Antibodies

Antibodies used

Human monoclonal anti-Ebola glycoprotein IgG antibody 9.20.1C3 ("1C3").

Anti-human/Rhesus Macaque antibodies used for flow cytometry staining are listed below:
Antibody, Company, Catalog #, Lot #

Antibodies and reagents for flow cytometry:
PBS, Gibco, 10010-049, 2053546
Ultrapure Water, Gibco, 10977-015, 2048083
10X BD Perm Wash, BD Biosciences, 51-2091KZ, 8088837
BD CytoFix CytoPerm, BD Bioscience, 51-2090KZ, 8206956
Sheath Fluid, BD Bioscience, 342003, 0000202111
Count Bright Beads, ThermoFisher, C36950, 2014179
Live/DEAD Fixable Aqua Dead Cell Stain Kit, Molecular Probes, L34957, 2008162
CD27 BV421, Biolegend, 356418, 8265880
CD14 BV570, Biolegend, 301832, 8245763
CD123 BV605, BD Biosciences, 564197, 8222563
IgD FITC, Southern Biotech, 2030-02, 02016-S277B
CD197(CCR7)PE, BD Biosciences, 560765, 9025622
CD8 BV650, Biolegend, 344730, 8274094
CD4 BV786, BD Biosciences, 563914, 8316948
CD20 AF700, BD Biosciences, 560631, 8215552
CD28 PerCP-Cy5.5, Biolegend, 302922, 8275635
CD45 PE-CF594, BD Biosciences, 562394, 8240808
CD95 PE-Cy5, Biolegend, 305610, B266014
CD3 PE-Cy7, BD Biosciences, 557749, 8208643
CD11c BV711, Biolegend, 301630, B278037
HLA-DR APC Fire750, Biolegend, 307658, B280512

Validation

Information previously published in "Asymmetric and non-stoichiometric glycoprotein recognition by two distinct antibodies results in broad protection against ebolaviruses" by Milligan et al., 2022. All flow cytometry antibodies from commercial vendors were validated by quality control testing before use, and where appropriate, they were titrated to determine the appropriate dilution factors that were used for staining and used per manufacturer's instructions.

Reference:
Milligan JC, Davis CW, Yu X, Ilinykh PA, Huang K, Halfmann PJ, Cross RW, Borisevich V, Agans KN, Geisbert JB, Chennareddy C, Goff AJ, Piper AE, Hui S, Shaffer KCL, Buck T, Heinrich ML, Branco LM, Crozier I, Holbrook MR, Kuhn JH, Kawaoka Y, Glass PJ, Bukreyev A, Geisbert TW, Worwa G, Ahmed R, Saphire EO. Asymmetric and non-stoichiometric glycoprotein recognition by two distinct antibodies results in broad protection against ebolaviruses. Cell. 2022 Mar 17;185(6):995-1007.e18. doi: 10.1016/j.cell.2022.02.023. PMID: 35303429.

Eukaryotic cell lines

Policy information about cell lines and Sex and Gender in Research

Cell line source(s)	VERO C1008 (E6) Kidney (African green monkey) cells. American Type Culture Collection (ATCC), Catalogue No. NR-596.
Authentication	Authentication was confirmed by isoenzyme analysis by the Charles River Laboratories, Inc., Malvern, PA.
Mycoplasma contamination	Mycoplasma contamination was not detected by Hoechst DNA stain, Agar and broth culture, and DNA detection by PCR.
Commonly misidentified lines (See ICLAC register)	N/A

Animals and other research organisms

Policy information about studies involving animals; ARRIVE guidelines recommended for reporting animal research, and Sex and Gender in Research

Laboratory animals	Rhesus monkey (Macaca mulatta; Zimmermann, 1780), Chinese origin.
Wild animals	Rhesus monkey was wild caught in China and imported through WorldWidePrimates, a commercial vendor. Animal was euthanized at the end of the study because it had been experimentally inoculated with Ebola virus. Live animals cannot be removed from Biosafety Level 4 maximum containment.
Reporting on sex	N/A
Field-collected samples	N/A
Ethics oversight	Experimental procedures involving the nonhuman primate (NHP) and infectious EBOV were conducted within the BSL-4 laboratory at the Integrated Research Facility at Fort Detrick (IRF-Frederick), National Institutes for Allergy and Infectious Diseases (NIAID), Division of Clinical Research (DCR), National Institutes of Health (NIH). The IRF-Frederick is accredited (000777) by the Association for Assessment and Accreditation of Laboratory Animal Care (AAALAC), approved for Laboratory Animal Welfare (D16-00602) by the Public Health Service (PHS), and registered (51-F-0016) with the United States Department of Agriculture (USDA). The study was approved by the NIAID DCR Animal Care and Use Committee (ACUC) under protocol IRF 033E and followed the recommendations provided in The Guide for the Care and Use of Laboratory Animals, the American Veterinary Medical Association (AVMA) guidelines for the euthanasia of animals.

Note that full information on the approval of the study protocol must also be provided in the manuscript.

Flow Cytometry

Plots

Confirm that:

☒ The axis labels state the marker and fluorochrome used (e.g. CD4-FITC).

☒ The axis scales are clearly visible. Include numbers along axes only for bottom left plot of group (a 'group' is an analysis of identical markers).

☒ All plots are contour plots with outliers or pseudocolor plots.

☒ A numerical value for number of cells or percentage (with statistics) is provided.

Methodology

Sample preparation	Fresh vitreous fluid was blocked with 5 µl of Human TruStain Fc Receptor Blocking Solution (Biolegend Cat#422302) on ice for 10 minutes followed by staining with a 100 µL master mix of antibodies in PBS on ice for 20 to 30 minutes. 50 µL of Count bright beads (ThermoFisher, Cat# C36950, Lot# 2014179) were added per sample for downstream enumeration of cell numbers. If applicable, red blood cells were lysed with 1 mL of 1x FACS Lyse per tube and incubated for 10 minutes at room temperature. Surface stained cells were washed with 3 mL of PBS-2% FBS-2mM EDTA (PBS-2), spun at 500 g for 5 minutes, supernatant was discarded and 500 µL of Cytofix/Cytoperm was added to each tube and incubated for 30 minutes at room temperature to inactivate and fix the cells. Fixed and inactivated cell samples were washed and spun as previously listed and samples were resuspended in 350 µL of PBS for flow cytometry acquisition.
Instrument	LSRII Fortessa instrument (BD Biosciences, Franklin Lakes, NJ, USA)
Software	BD FACS Diva software version 6.1.3 (BD Biosciences, Franklin Lakes, NJ, USA) was used for collection of raw flow cytometry data. FlowJo software version 10 (FlowJo, Ashland, OR 97520, USA) was used for analysis of flow cytometry data.
Cell population abundance	No post-sort fractions were collected in this study.
Gating strategy	FSC-A/FSC-H and SSC-W/SSC-A gating was used to identify singlets. Viability dye was used to exclude dead cells. Live cells were gated for CD45 positivity and plotted on FSC-A/SSC-A to identify CD45+ cells of the appropriate size. CD3+ cells were further divided into CD8+ T cells or CD4+ T cells. CD4+ and CD8+ T cells were further identified as naive (CD28+/CD95-), central memory (CD28+/CD95+), or effector memory (CD28-, CD95+) populations. Of the live CD45+ cells, B cells were not observed, but characterized as naive (CD20+ CD3- CD27- IgD+), Marginal-zone (CD20+ CD3- CD27+ IgD+), or Memory B cells (CD20+ CD3- CD27+ IgD-). Cells within the CD45+ CD20- CD3- gate were divided into Monocytes (CD14+), Plasmacytoid Dendritic cells (CD14- HLA-DR+ CD123+) or Myeloid Dendritic Cells (CD14- HLA-DR+ CD123- CD11c+).

☒ Tick this box to confirm that a figure exemplifying the gating strategy is provided in the Supplementary Information.

Magnetic resonance imaging

Experimental design

Design type	Pre-post contrast T1 weighted and *pre-contrast T2W and STIR imaging of the orbits
Design specifications	N/A
Behavioral performance measures	N/A

Acquisition

Imaging type(s)	T1W pre/post contrast, pre contrast T2W and STIR
Field strength	3T
Sequence & imaging parameters	Axial T1W pre/post contrast: spin echo (SE); 2D, repetition time (TR), 600 ms; echo time (TE), 15 ms; flip angle (FA), 90 degrees; slice thickness, 2 mm; 10% gap; acquired in-plane resolution, 0.8 mm x 0.8 mm.<br><br>Sagittal T1W pre/post contrast: spin echo (SE); 2D; TR, 675 ms; TE, 15 ms; flip angle (FA), 90 degrees; slice thickness, 2 mm; 10% gap; acquired in-plane resolution, 0.8 mm x 0.8 mm.<br><br>Coronal SPIR pre-contrast: spin echo (SE) inversion recovery (IR); 2D; TR, 3000 ms; TE, 60 ms; inversion time (TI), 200 ms; flip angle (FA), 90 degrees; slice thickness, 2 mm; 0% ap; acquired in-plane resolution, 0.8 mm x 0.8 mm.<br><br>Axial T2W pre/post contrast: spin echo (SE); 2D; TR, 3600 ms; TE, 100 ms; flip angle (FA), 90 degrees; slice thickness, 1.3 mm; 0% gap; acquired in-plane resolution, 0.7 mm x 0.7 mm.
Area of acquisition	*State whether a whole brain scan was used OR define the area of acquisition, describing how the region was determined.*

Diffusion MRI ☐ Used ☒ Not used

Preprocessing

Preprocessing software	N/A
Normalization	N/A
Normalization template	N/A
Noise and artifact removal	N/A
Volume censoring	N/A

Statistical modeling & inference

Model type and settings	N/A
Effect(s) tested	N/A

Specify type of analysis: ☐ Whole brain ☐ ROI-based ☐ Both

Statistic type for inference (See Eklund et al. 2016)	N/A
Correction	N/A

Models & analysis

n/a	Involved in the study
☒	☐ Functional and/or effective connectivity
☒	☐ Graph analysis
☒	☐ Multivariate modeling or predictive analysis
